# Metformin suppresses PPARδ-driven CD47 transcription to enhance macrophage phagocytosis in lung cancer

**DOI:** 10.1016/j.jbc.2026.111159

**Published:** 2026-01-13

**Authors:** Qian Cui, Huijie Wang, Hongxu Yu, Yinghan Li, Yelei Wang, Juanjuan Shi, Yongzhong Hou

**Affiliations:** School of Life Science, Jiangsu University, Zhenjiang, Jiangsu Province, People's Republic of China

**Keywords:** metformin, CD47, PPARδ, phagocytosis, immune evasion

## Abstract

Metformin, an activator of AMP kinase, influences critical cellular processes, including proliferation, metabolism, inflammation, and immunity. However, its specific impact on macrophage-mediated phagocytosis of tumor cells remains poorly characterized. Our study demonstrates that metformin treatment substantially decreases both CD47 protein and mRNA levels in lung cancer cells. This reduction stems from metformin's suppression of CD47 gene transcription. Consequently, metformin enhances macrophage phagocytic activity against cancer cells. *In vivo* analyses using a tumor implantation model revealed that metformin impedes tumor immune escape. This effect correlates with diminished CD47 expression within tumors and heightened macrophage phagocytosis. Furthermore, combining metformin with an anti-CD47 antibody synergistically augmented antitumor immunotherapy efficacy. Mechanistically, metformin attenuates peroxisome proliferator–activated receptor delta–mediated CD47 transcriptional activation and subsequent gene expression. These results elucidate a novel mechanism by which metformin counteracts tumor immune evasion.

Metformin, a synthetic biguanide, is widely regarded as a foundational therapy for type 2 diabetes and ranks among the most commonly prescribed medications worldwide (1). In addition to its well-established glucose-lowering effects, metformin exhibits a significant role in protection against tumor development (2, 3). Clinically, metformin use is associated with reduced cancer incidence and improved outcomes in colorectal, pancreatic, hepatocellular, breast, and lung cancer (4, 5, 6, 7), underscoring its potential as an antineoplastic agent. The AMP-activated protein kinase (AMPK) serves as a primary target of metformin, functioning as a central sensor of cellular energy status (8). Metformin-induced AMPK activation restrains cancer cell proliferation and tumor growth (9, 10, 11, 12, 13, 14). Importantly, metformin also modulates tumor immunology. It can reduce programmed cell death ligand 1 (PD-L1) expression on cancer cells, thereby augmenting T cell–mediated cytotoxicity (15, 16). This positions metformin as a modulator of cancer immune evasion, although its full immunomodulatory potential remains incompletely characterized.

Cancer immune evasion is orchestrated through multiple, nonredundant checkpoint systems. While the programmed cell death protein 1 (PD-1)–PD-L1 axis constitutes a dominant “don't find me” signal impairing adaptive T-cell immunity, the CD47–signal regulatory protein α (SIRPα) axis represents a crucial “don't eat me” signal that dampens innate immune responses (15, 17, 18, 19, 20). CD47, frequently overexpressed in lung cancer and other solid tumors, binds to SIRPα on macrophages, thereby suppressing phagocytic clearance and facilitating immune escape (17, 21, 22, 23, 24, 25). The transcriptional regulation of CD47 is governed by several factors, including Myc, NF-κB, hypoxia-inducible factor 1, and nuclear respiratory factor 1 (26, 27, 28, 29). Notably, the nuclear receptor peroxisome proliferator–activated receptor delta (PPARδ), encoded by the *PPARD* gene, has been identified as a direct transcriptional activator of CD47. Ligand-mediated activation of PPARδ leads to increased CD47 expression, which consequently inhibits macrophage phagocytosis (21). PPARδ activity is itself regulated by AMPK (30, 31, 32, 33, 34). Specifically, AMPK can directly bind to PPARδ and induce its phosphorylation, an action that inhibits PPARδ′s transcriptional activity and suppresses colorectal cancer cell proliferation. Furthermore, the AMPK agonist metformin enhances this inhibitory process (13, 35). Although PPARδ is established as a transcriptional activator of CD47 (21), the potential involvement of the AMPK–PPARδ axis in CD47-mediated immune escape remains unexplored. Here, we found that metformin–AMPK–PPARδ pathway inhibited CD47-mediated tumor immune escape.

## Results

### Metformin suppresses CD47 membrane expression

**Figure 1:**
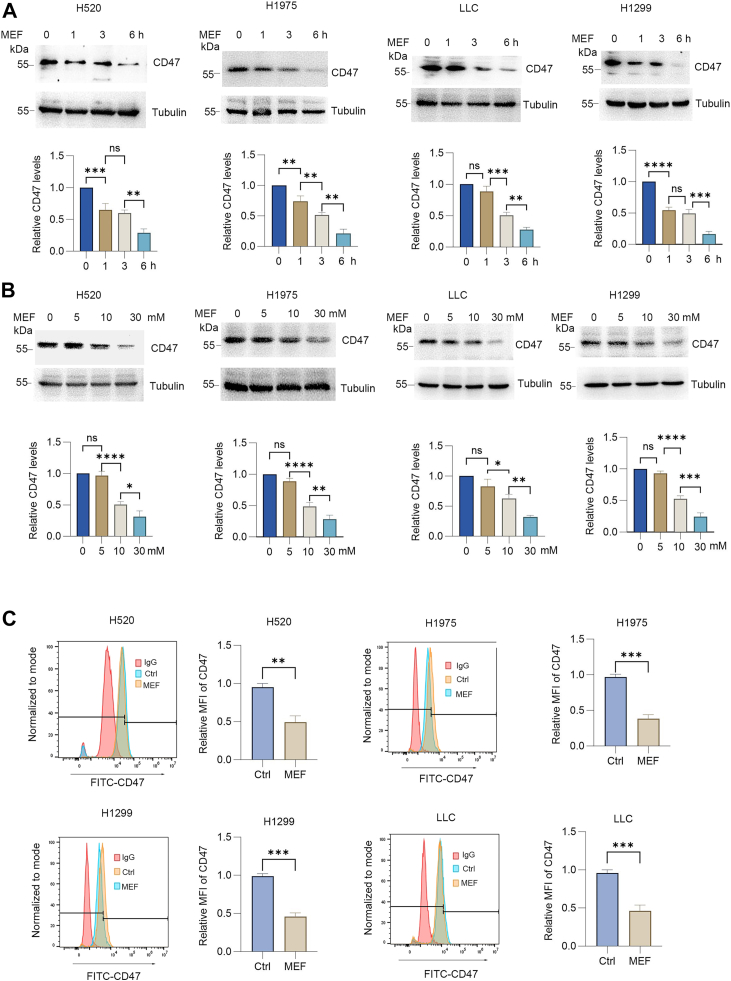
**Metformin reduces CD47 protein expression.***A*, cells were treated with 30 mM metformin for the indicated times. Western blot analysis was performed, and the blots were quantified (mean ± SD, n = 3). *B*, cells were treated with metformin (0, 5, 10, and 30 mM) for 6 h. Cell lysates were analyzed by Western blot, and blots were quantified (mean ± SD, n = 3). *C*, surface CD47 expression was analyzed by flow cytometry in cells treated with 30 mM metformin for 6 h. Median fluorescence intensity (MFI) values are shown as mean ± SD (n = 3). A paired Student's t test was used for comparison between two related groups. One-way ANOVA or two-way ANOVA was selected based on the number of independent variables in the experimental design for comparison among three or more groups: statistical significance thresholds were set at p < 0.05, ∗*p* < 0.05, ∗∗*p* < 0.01, ∗∗∗<0.001,∗∗∗∗<0.0001.

**Figure 2:**
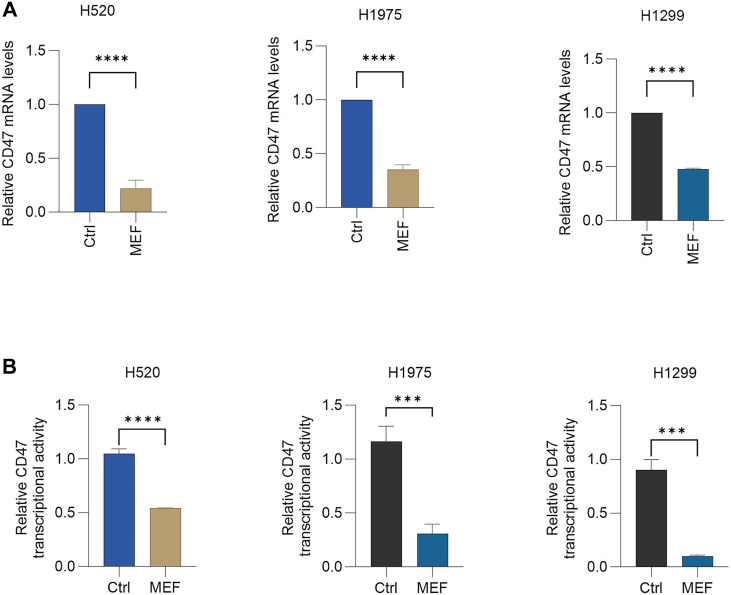
**Metformin inhibits CD47 expression at the transcriptional level.***A*, quantitative PCR analysis showing reduced CD47 mRNA levels in cells treated with 30 mM metformin for 6 h. Data represent mean ± SD (n = 3). *B*, luciferase reporter assay of CD47 promoter activity. Cells cotransfected with pGL3-CD47-lu and Ptk-RL plasmids for 48 h were treated with 30 mM metformin for 6 h. Data represent mean ± SD (n = 3). A paired Student's t test was used for comparison between two related groups. One-way ANOVA or two-way ANOVA was selected based on the number of independent variables in the experimental design for comparison among three or more groups: statistical significance thresholds were set at *p* < 0.05, ∗*p* < 0.05, ∗∗*p* < 0.01, ∗∗∗<0.001,∗∗∗∗<0.0001.

To evaluate metformin's effect on CD47 regulation, four lung cancer cell lines (H520, H1975, H1299, and Lewis lung carcinoma [LLC]) were treated with 30 mM metformin for different durations. Immunoblotting analysis demonstrated a time-dependent attenuation of total CD47 protein (Fig. 1*A*). Dose–course experiments further established progressive CD47 reduction over treatment duration (Fig. 1*B*). Flow cytometric quantification confirmed a significant reduction of membrane-localized CD47 in all tested cell lines (Fig. 2*C*). These findings collectively indicate that metformin, as an AMPK activator, downregulates surface CD47 expression in lung cancer cells.

### Metformin transcriptionally represses CD47 expression

Building on the observed CD47 protein suppression, we investigated transcriptional regulation as a potential mechanism. Quantitative PCR analysis revealed a decrease in CD47 mRNA levels in lung cancer cells (H520, H1975, H1299, and LLC), following treatment with 30 mM metformin (Fig. 2*A*). This transcriptional inhibition was further corroborated by luciferase reporter assays, showing significant attenuation of CD47 promoter activity following metformin exposure (Fig. 2*B*). Collectively, these data establish transcriptional repression as the primary mechanism through which metformin downregulates CD47 expression.

### Metformin potentiates macrophage phagocytic function

**Figure 3:**
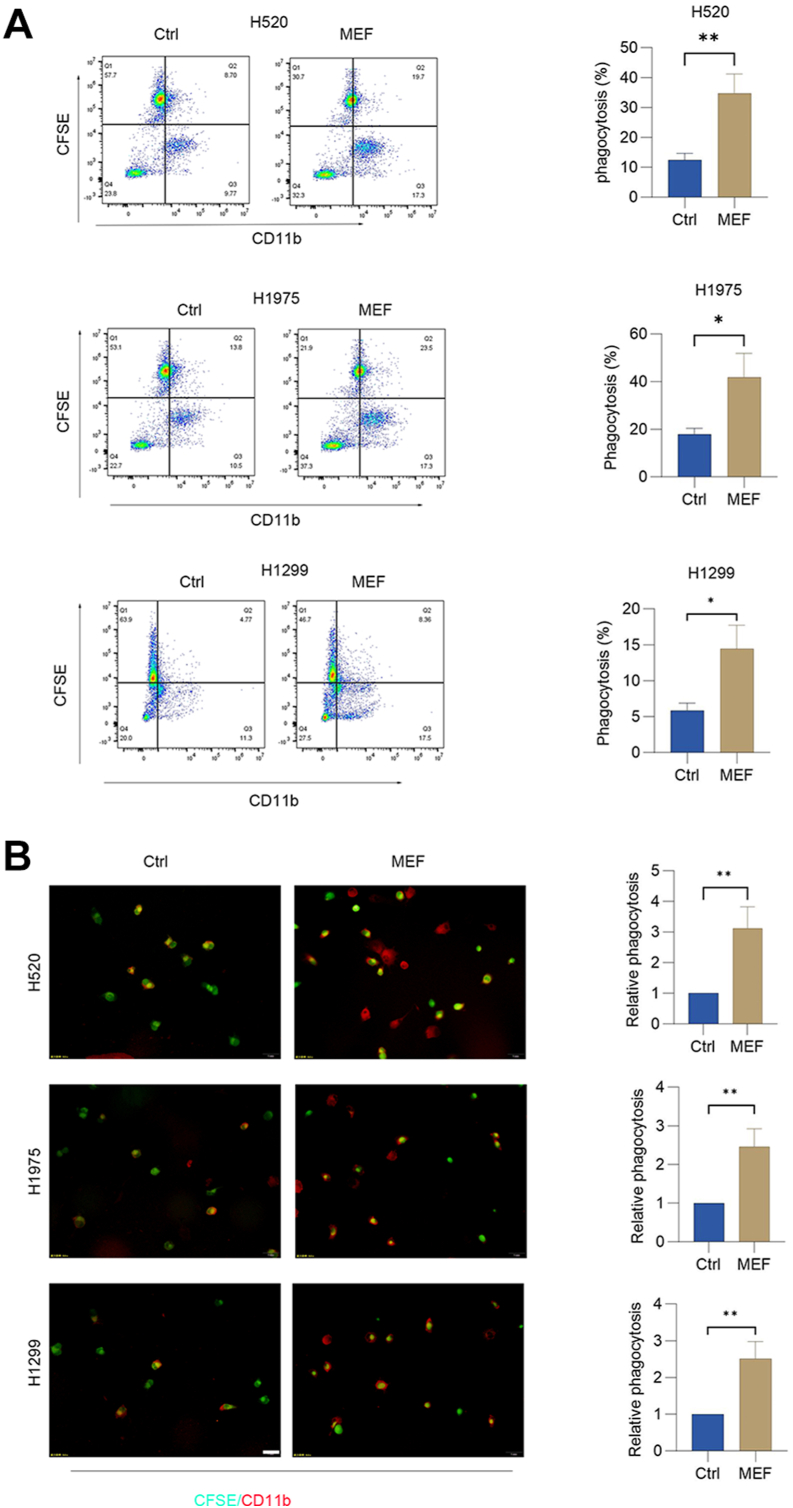
**Metformin potentiates macrophage phagocytic function.***A*, flow cytometry analysis of phagocytosis in macrophages cocultured with target cells preexposed to 30 mM metformin or control (6 h). The percent of phagocytosis was quantified. Data represent mean ± SD (n = 3). *B*, CFSE-labeled target cells and anti–CD11b-stained macrophages were analyzed after coculture with metformin-treated targets (30 mM, 6 h). Phagocytic activity was assayed by immunofluorescence. The scale bars represent 50 μm. More than 100 cells were scored (mean ± SD, n = 4). A paired Student's t test was used for comparison between two related groups. One-way ANOVA or two-way ANOVA was selected based on the number of independent variables in the experimental design for comparison among three or more groups: statistical significance thresholds were set at *p* < 0.05, ∗*p* < 0.05, ∗∗*p* < 0.01, ∗∗∗<0.001,∗∗∗∗<0.0001. CFSE, carboxyfluorescein succinimidyl ester.

The CD47–SIRPα checkpoint axis suppresses macrophage-mediated phagocytosis by transmitting “don't eat me” signals (22). Given our findings that metformin transcriptionally represses CD47 in lung cancer cells, we investigated its functional impact on immune evasion. To assess whether CD47 downregulation reverses phagocytic inhibition, macrophages were cocultured with lung cancer cells pretreated with metformin. Quantitative phagocytosis assays revealed an increase in macrophage engulfment of metformin-exposed tumor cells *versus* controls (Fig. 3, *A* and *B*). This demonstrates that metformin-induced CD47 suppression functionally antagonizes the CD47–SIRPα axis, thereby enhancing immunophagocytic clearance of lung cancer cells.

### Metformin inhibits tumor immune evasion *via* suppression of CD47

**Figure 4:**
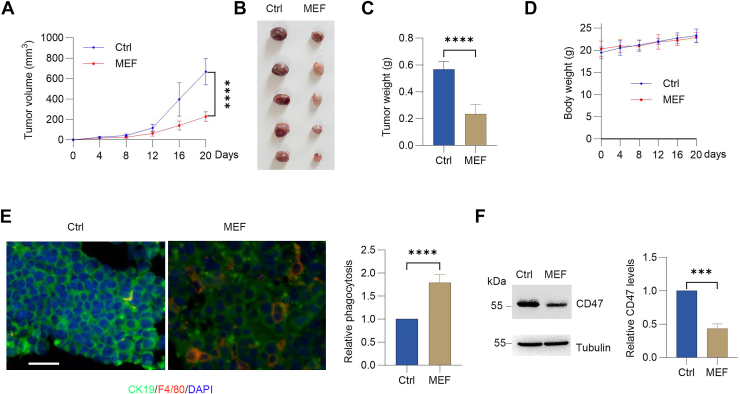
**Metformin blocks tumor immune escape *in vivo*.***A*–*D*, subcutaneous LLC tumor–bearing C57BL/6 mice received metformin treatment. Tumor volume, tumor weight, and body weight were measured (mean ± SD; n = 5). *E*, multiplex immunofluorescence staining of tumor sections showing CK19 (tumor) and F4/80 (macrophage) levels. The scale bars represent 20 μm. Phagocytic activity was assayed by immunofluorescence. More than 100 cells were scored (mean ± SD; n = 5). *F*, CD47 protein levels in tumor lysates by immunoblotting with quantitative analysis (mean ± SD; n = 5). A paired Student's t test was used for comparison between two related groups. One-way ANOVA or two-way ANOVA was selected based on the number of independent variables in the experimental design for comparison among three or more groups: statistical significance thresholds were set at *p* < 0.05, ∗*p* < 0.05, ∗∗*p* < 0.01, ∗∗∗<0.001,∗∗∗∗<0.0001. LLC, Lewis lung carcinoma.

Cancer cells evade macrophage phagocytosis by expressing CD47 (22). To assess whether metformin counteracts this immune evasion mechanism by reducing CD47 levels, we utilized a subcutaneous murine lung tumor model. Compared with saline-treated controls, metformin-treated mice exhibited significantly slower tumor progression and lower terminal tumor mass (Fig. 4, *A* and *B*). Analysis of tumors by Western blot demonstrated that metformin administration effectively lowered CD47 expression (Fig. 4*C*). Concurrently, we observed a substantial increase in F4/80^+^ macrophage infiltration within the tumors (Fig. 4, *D* and *E*), which was associated with heightened phagocytic activity.

### Metformin exhibits additive effects with the CD47 antibody on antitumor immunity

**Figure 5:**
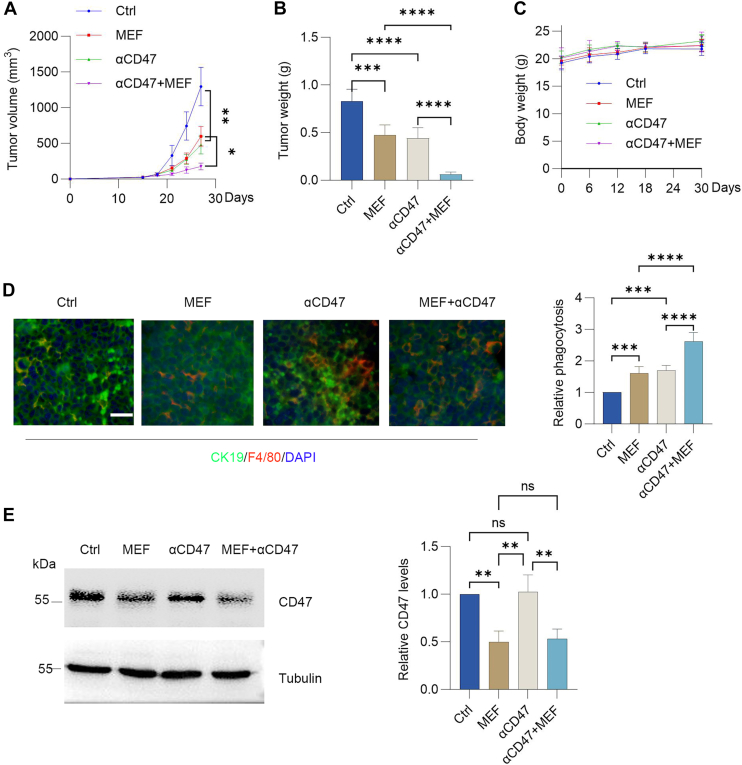
**Metformin exhibits additive effects with the CD47 antibody on antitumor immunity.***A*–*C*, C57BL/6 mice with subcutaneously implanted LLC cells (2 × 10^5^) received metformin monotherapy, anti-CD47 monotherapy, or combination therapy. Tumor volume, tumor weight, and body weight were measured (mean ± SD; n = 5). *D*, multiplex immunofluorescence staining of tumor sections showing CK19 (tumor) and F4/80 (macrophage) levels. The scale bars represent 20 μm. Phagocytic activity was assayed by immunofluorescence. More than 100 cells were scored (mean ± SD; n = 5). *E*, immunoblot-based quantification of CD47 protein in tumor lysates (mean ± SD; n = 5). A paired Student's t test was used for comparison between two related groups. One-way ANOVA or two-way ANOVA was selected based on the number of independent variables in the experimental design for comparison among three or more groups: statistical significance thresholds were set at *p* < 0.05, ∗*p* < 0.05, ∗∗*p* < 0.01, ∗∗∗<0.001,∗∗∗∗<0.0001. LLC, Lewis lung carcinoma.

Capitalizing on the observation that metformin impedes tumor immune evasion, we explored its capacity to enhance the effectiveness of immunotherapy. In a murine tumor model, animals were treated with metformin alone, anti-CD47 antibody alone, or both agents combined. Metformin monotherapy curbed tumor growth and reduced the end-point tumor mass. However, the combined regimen achieved significantly greater suppression of tumor progression and final mass than either single-agent treatment (Fig. 5, *A* and *B*). Immunofluorescence assessment further showed that tumors from the combination group had markedly increased infiltration of F4/80^+^ macrophages compared with those treated solely with anti-CD47 antibody (Fig. 5*C*). These findings indicate that metformin exhibits additive effects with the CD47 antibody on antitumor immunity.

### Metformin suppresses CD47 expression dependent on PPARδ

**Figure 6:**
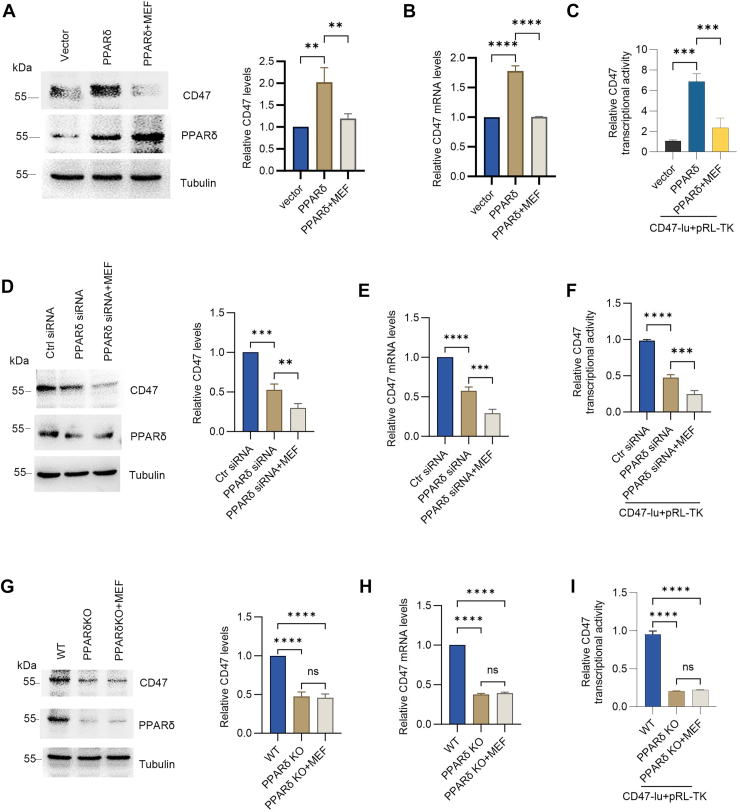
**Metformin downregulates CD47 *via* a PPARδ-dependent pathway.***A*, H520 cells were transfected with pcDNA3 (control) or pcDNA3-FLAG-PPARδ. After 48 h, cells were treated with or without 30 mM metformin for 6 h. Western blot analysis and blot quantification were performed (mean ± SD, n = 3). *B*, H520 cells were transfected with pcDNA3 (control) or pcDNA3-FLAG-PPARδ. After 48 h, cells were treated with or without 30 mM metformin for 6 h. Quantitative PCR analysis was performed (mean ± SD, n = 3). *C*, H520 cells transfected with the indicated plasmids were exposed to vehicle or 30 mM metformin for 6 h. Luciferase reporter assay was performed (mean ± SD, n = 3). *D*, H520 cells were transfected with control siRNA or PPARδ siRNA. After 48 h, cells were treated with or without 30 mM metformin for 6 h. Western blot and quantification were performed (mean ± SD, n = 3). *E*, H520 cells were transfected with control siRNA or PPARδ siRNA. After 48 h, cells were treated with or without 30 mM metformin for 6 h. Quantitative PCR analysis was performed (mean ± SD, n = 3). *F*, H520 cells were transfected with control siRNA or PPARδ siRNA together with reporter plasmids as indicated. After 48 h, cells were treated with or without 30 mM metformin for 6 h. Luciferase reporter assay was performed (mean ± SD, n = 3). *G*, WT and PPARδ-knockout H520 cells were treated with 30 mM metformin for 6 h. Western blot and quantification were performed (mean ± SD, n = 3). *H*, WT and PPARδ-knockout H520 cells were treated with 30 mM metformin for 6 h. Quantitative PCR analysis was performed (mean ± SD, n = 3). *I*, WT and PPARδ-knockout H520 cells transfected with reporter plasmids as indicated were treated with or without 30 mM metformin for 6 h after 48 h. Luciferase reporter assay was performed (mean ± SD, n = 3). A paired Student's t test was used for comparison between two related groups. One-way ANOVA or two-way ANOVA was selected based on the number of independent variables in the experimental design for comparison among three or more groups: statistical significance thresholds were set at *p* < 0.05, ∗*p* < 0.05, ∗∗*p* < 0.01, ∗∗∗<0.001,∗∗∗∗<0.0001. PPARδ, peroxisome proliferator–activated receptor delta.

Our previous investigation suggested that PPARδ induces CD47 expression (36). Metformin downregulates CD47 expression, but its dependence on PPARδ remained undetermined. To investigate this mechanism, we overexpressed PPARδ in H520 cells and treated them with metformin. Western blot analysis showed that overexpressed PPARδ increased CD47 expression, which was reversed by metformin (Fig. 6*A*). Similarly, overexpressed PPARδ increased CD47 gene expression and transcriptional activity, which was reduced by metformin treatment (Fig. 6, *B* and *C*). Metformin treatment had a more significant effect (Fig. 6, *D*–*F*, Fig S1), which was consistent with the results from PPARδ-knockout H520 cells generated by CRISPR–Cas9 (Fig. 6, *G*–*I*). These findings suggest that metformin decreases CD47 expression in a PPARδ-dependent manner.

### Metformin inhibits CD47 expression *via* the AMPK–PPARδ pathway

**Figure 7:**
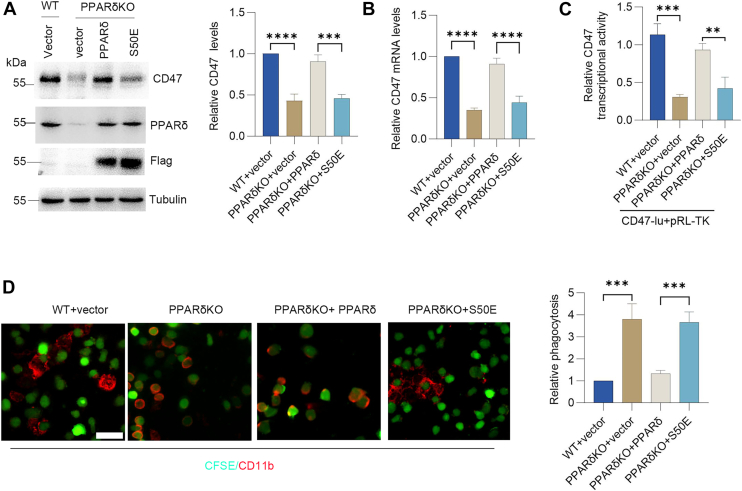
**PPARδ phosphomimetic mutant (S50E) loses CD47 induction capacity.***A*, Western blot analysis of CD47 protein expression in WT or PPARδ-deficient (PPARδ^−/−^) H520 cells re-expressing either PPARδ or the S50E mutant. Quantified CD47 protein levels are shown as mean ± SD (n = 3). *B*, quantitative PCR analysis of CD47 mRNA levels in WT or PPARδ^−/−^ H520 cells re-expressing PPARδ or the S50E mutant. Data are presented as mean ± SD (n = 3). *C*, luciferase reporter assay measuring CD47 promoter activity in WT or PPARδ^−/−^ H520 cells re-expressing PPARδ or the S50E mutant. Data are presented as mean ± SD (n = 3). *D*, phagocytosis assay: CFSE-labeled WT or PPARδ^−/−^ H520 cells re-expressing PPARδ or the S50E mutant were cocultured with macrophages (CD11b). Phagocytic activity was assayed by immunofluorescence. The scale bars represent 50 μm. More than 100 cells were scored; data are presented as mean ± SD (n = 4). A paired Student's t test was used for comparison between two related groups. One-way ANOVA or two-way ANOVA was selected based on the number of independent variables in the experimental design for comparison among three or more groups: statistical significance thresholds were set at *p* < 0.05, ∗*p* < 0.05, ∗∗*p* < 0.01, ∗∗∗<0.001,∗∗∗∗<0.0001. CFSE, carboxyfluorescein succinimidyl ester; PPARδ, peroxisome proliferator–activated receptor delta.

Previous investigations show that metformin facilitates AMPK-mediated phosphorylation of the PPARδ S50 residue, leading to inhibition of PPARδ activity (13). To further determine the effect of the PPARδ phosphomimetic mutant (S50E) on CD47 expression, PPARδ-deficient (PPARδ^−/−^) H520 cells were reconstituted with either WT PPARδ or the S50E mutant. The results showed that although overexpression of PPARδ increased CD47 expression, the PPARδ–S50E mutant reversed this effect (Fig. 7*A*). Similarly, PPARδ promoted CD47 gene expression and increased its transcriptional activity, whereas the PPARδ–S50E mutant reversed this effect (Fig. 7, *B* and *C*). To assess the effect of the PPARδ–S50E mutant on macrophage phagocytosis, macrophages were cocultured with H520 cells that had been reconstituted with either WT PPARδ or the S50E mutant. The results revealed that overexpression of PPARδ inhibited phagocytosis, but the S50E mutant reversed this effect (Fig. 7*D*). These findings suggest that metformin inhibits CD47 expression *via* the AMPK–PPARδ pathway.

## Discussion

The host immune system possesses inherent antitumor capabilities when activated (37, 38). However, the immune checkpoint protein PD-L1, acting as a “don't find me” signal, binds PD-1 on T cells to promote immunosuppression in cancer (18, 19, 39). Beyond the established role of adaptive immune checkpoints like PD-1–PD-L1 in cancer immunotherapy, innate immune checkpoints, particularly the SIRPα–CD47 axis, are crucial regulators of tumor immune surveillance (22). CD47, a glycoprotein frequently overexpressed on cancer cells, engages SIRPα on phagocytes. This interaction delivers an inhibitory “don't eat me” signal, suppressing phagocytosis (40, 41). The elevated CD47 levels observed in tumors are transcriptionally regulated by factors, including NF-κB, HIF, Myc, and PPARδ (26, 28, 36, 42, 43). Consequently, reducing CD47 expression disrupts this signal, enhancing macrophage phagocytosis of cancer cells and thereby inhibiting tumor immune escape (21, 36). Metformin—primarily used for diabetes—is accumulating evidence for exhibiting anticancer effects across various malignancies (44), alongside targeted therapies and immunotherapy (45). Metformin activates AMPK, which subsequently triggers phosphorylation of PD-L1 at serine-195. This phosphorylation impairs PD-L1 glycosylation and targets it for endoplasmic reticulum–associated protein degradation (16). Consequently, metformin augments antitumor immunotherapy efficacy (46, 47). In this study, our results demonstrated that treatment with the AMPK agonist metformin significantly decreased CD47 expression. This decrease, in turn, increased macrophage phagocytosis of cancer cells. This study revealed a novel role of metformin on the macrophage phagocytosis in the tumor microenvironment by reducing CD47 expression.

Numerous studies suggest that metformin treatment affects T-cell activity by reprogramming tumor-specific T-cell metabolism (48) and reducing PD-L1 expression (16). This consequently enhances the host adaptive immunity, which in turn blocks tumor immune escape and effectively enhances antitumor immunotherapy (16, 46, 47, 48). Our study also provides insight into the effect of metformin on innate immune checkpoints (the SIRPα–CD47 axis).

PPARδ, a member of the PPAR family (49, 50), is activated by fatty acids and their derivatives. This receptor participates in mucosal inflammation and malignant transformation (51) with aberrant expression or activation driving metastatic progression and tumorigenesis (51, 52, 53, 54, 55, 56, 57, 58, 59). Upon activation, PPARδ binds peroxisome proliferator response elements in target gene promoters to regulate transcription (60, 61, 62, 63). AMPK, a conserved metabolic sensor in eukaryotes, responds to ATP depletion by modulating cellular and organismal metabolism (11, 64), critically regulating cell growth and metabolic pathways (11). Metformin activates AMPK, which phosphorylates PPARδ. This phosphorylation event inhibits PPARδ′s transcriptional activity—reducing glucose/glutamine uptake and suppressing cancer cell proliferation and tumor growth (13). Notably, our prior work demonstrated that PPARδ activation upregulates CD47 transcription, inhibiting macrophage phagocytosis (36). Conversely, PPARδ antagonism suppresses CD47 expression, promoting phagocytosis and limiting tumor immune escape (21). In this study, PPARδ overexpression elevated CD47 levels—an effect abrogated by metformin. Similarly, PPARδ overexpression inhibited phagocytosis, whereas the phospho-mimicking mutant PPARδ–S50E failed to upregulate CD47. These findings align with AMPK-mediated phosphorylation of PPARδ at Ser50, suppressing its transcriptional function (13) and suggest that metformin decreases CD47 expression in a PPARδ-dependent manner.

In conclusion, this study demonstrates that metformin treatment enhances macrophage phagocytosis by reducing CD47 expression in a PPARδ-dependent manner in lung cancer cells, thereby inhibiting tumor immune escape. These findings reveal a potential immunotherapy strategy.

## Experimental procedures

### Antibodies, reagents, and plasmids

The primary antibodies recognizing tubulin, PPARδ, CK19, and CD47 were derived from Proteintech. PE-labeled CD11b and FITC-labeled CD47 antibodies, as well as matching PE-IgG and FITC-IgG isotype controls, were all obtained from Elabscience. Jackson ImmunoResearch provided the secondary antibodies. PPARδ siRNA was purchased from Sangon Biotec. Sense: A1GUUUGAAUUUGCUGUCAAGU; antisense: UUGACAGCAAAUUCAAACUUA. In addition, the metformin (MedChemExpress) and a protease inhibitor cocktail (Yeasen) were used. PPARδ and PPARδ–S50E were described previously (13). pGl3-CD47-lu, pRL-TK plasmids were described previously (36).

### Cell culture

The human cancer cell lines utilized in this study (H1975, H1299, H520, and THP-1) and the murine cancer cell line LLC were procured from the National Collection of Authenticated Cell Cultures. PPARδ knockout H520 cells were described (36). Prior to experimentation, each line underwent authentication *via* short tandem repeat analysis and was verified as mycoplasma negative. Cultivation media consisted of RPMI1640 for THP-1 and LLC cells, and Dulbecco's modified Eagle's medium for H1975, H520, and H1299 cells. All media formulations contained 10% fetal bovine serum (Gibco). Plasmid transfections were performed using polyethylenimine.

### Western blot analysis

Cells were washed twice with ice-cold PBS and centrifuged to form pellets. Cell lysis was conducted using a buffer composed of 50 mM Tris–HCl (pH 7.4), 250 mM NaCl, 0.5% Triton X-100, 10% glycerol, 1 mM DTT, and protease inhibitor cocktail. After centrifugation to clarify lysates, supernatants were harvested. Equal protein quantities were separated by SDS-PAGE and subsequently transferred electrophoretically onto nitrocellulose membranes. Membranes were probed with primary antibodies overnight at 4 °C. Following three washes with PBS with Tween-20, horseradish peroxidase–conjugated anti-mouse/rabbit, immunoreactive bands were visualized by enhanced chemiluminescence substrate exposure, and chemiluminescent signals were recorded. Quantitative analysis of band intensities was carried out with ImageJ (NIH) software.

### Analysis of surface CD47 levels by flow cytometry

Cells were treated with either dimethyl sulfoxide (vehicle control) or 30 mM metformin for 6 h. Post-treatment, cells were washed three times with PBS and subjected to surface staining using FITC-conjugated anti-CD47 antibody with parallel isotype control (IgG) on ice for 1 h. After three additional PBS washes to remove unbound antibodies, cellular fluorescence was quantified on a CytoFLEX flow cytometer.

### Immunofluorescence

Tumor specimens from mice were fixed by immersion in 10% neutral-buffered formalin. After paraffin embedding, consecutive 5 μm tissue sections were cut. Deparaffinized sections underwent heat-induced antigen retrieval in sodium citrate buffer (10 mM sodium citrate, 0.05% Tween-20, pH 6.0) for 20 min to unmask epitopes. Following three PBS washes, nonspecific binding sites were blocked with 1% bovine serum albumin in PBS. Primary antibodies targeting CK19 or F4/80 were applied for overnight immunostaining at 4 °C. Sections were then incubated with fluorescence-conjugated secondary antibodies. Finally, antibody-labeled tissues were imaged under a fluorescence microscope. The phagocytic index, defined as the number of ingested CK19-labeled cancer cells per 100 F4/80-positive macrophages, was quantified using ImageJ.

### Quantitative real-time PCR analysis

H1975, H520, and H1299 cells were treated with 30 mM metformin for 6 h. Total RNA was extracted and reverse transcribed into complementary DNA using HiScript III RT SuperMix (Vazyme). CD47 mRNA expression was quantified with ChamQ Universal SYBR qPCR Master Mix (Vazyme), normalized to β-actin as endogenous control. Relative gene expression *versus* dimethyl sulfoxide vehicle control was calculated *via* the 2^-ΔΔCt^ method.

### Phagocytosis assay

THP-1 monocytes were polarized into macrophages by 5-day incubation with 100 ng/ml phorbol 12-myristate 13-acetate. Cancer cells were fluorescently labeled with carboxyfluorescein succinimidyl ester (CFSE; Solarbio) and washed three times with PBS. Labeled cancer cells (4 × 10^5^) were cocultured with macrophages (1 × 10^5^) at a 4:1 effector-to-target ratio in serum-free medium for 4 h to assess engulfment capacity. For phagocytosis analysis by flow cytometry, harvested cells were labeled with CD11b antibody (1:500 dilution) and subjected to flow cytometry. The phagocytosis percentage was defined as the proportion of CD11b^+^CFSE^+^ cells (Q1) among all CFSE^+^ cancer cells (Q1 + Q2). In the fluorescence microscopy assay, cells were similarly stained with CD11b antibody (1:500 dilution) and visualized under a fluorescence microscope. The phagocytic index, defined as the number of ingested CFSE-labeled cancer cells per 100 CD11b-positive macrophages, was quantified using ImageJ.

### Luciferase reporter assay for CD47 transcriptional activity

To assess CD47 promoter activity, cells were cotransfected with a CD47-luciferase reporter plasmid (pGL3-CD47-luc) and a Renilla luciferase control plasmid (pTK-RL) for 48 h. Following transfection, the cells were treated with metformin. In a separate experiment, the same plasmids were transfected into WT or PPARδ^−/−^ H520 cells that were re-expressing either PPARδ or the S50E mutant. Subsequently, cell lysates were prepared and analyzed using a dual-luciferase reporter assay kit (GN201-01; YPH Biotech) following the manufacturer's protocol. Firefly luciferase activity was normalized to Renilla luciferase activity for each sample to determine relative CD47 promoter–driven transcription.

### Subcutaneous tumor xenograft model

To establish tumor xenografts, male C57BL/6 mice (5 weeks old) were subcutaneously inoculated with LLC cells (1 × 10^5^ cells per mouse). Following tumor engraftment after 7 days, mice were randomly assigned to four treatment groups (n = 5 per group): group 1 (vehicle): received vehicle control (saline); group 2 (metformin): administered metformin (10 mg/kg/day) *via* intraperitoneal injection; group 3 (anti-CD47): treated with anti-CD47 monoclonal antibody (100 μg per mouse) *via* intratumoral injection; and group 4 (combination): Received both metformin and anti-CD47 monoclonal antibody at the doses specified above. Tumor size was monitored regularly using digital calipers. Tumor volume was calculated using the formula: volume (mm^3^) = 0.5 × length × width^2^. All animal experiments were conducted in accordance with protocols approved by the Jiangsu University Animal Care Committee (approval no.: 20180053). Mice were obtained from the Animal Center of Jiangsu University.

### Statistical analysis

Data analysis was performed using GraphPad Prism software (Dotmatics, version 10.1.2). Continuous variables are presented as mean ± SD. Statistical comparisons were conducted as follows:

A paired Student's *t* test was used for comparisons between two related groups. One-way ANOVA or two-way ANOVA was selected based on the number of independent variables in the experimental design for comparisons among three or more groups: statistical significance thresholds were set at *p* < 0.05, ∗*p* < 0.01, and ∗∗*p* < 0.001.

## Data availability

All data will be shared upon request to houyz@ujs.edu.com.

## Supporting information

This article contains supporting information.

## Conflict of interest

The authors declare that they have no conflicts of interest with the contents of this article.
