# Supplementary figures and images for: Metformin suppresses PPARδ-driven CD47 transcription to enhance macrophage phagocytosis in lung cancer
Source: J Biol Chem. 2026 Jan 13;302(3):111159. doi: 10.1016/j.jbc.2026.111159 (PMC12906186; doi:10.1016/j.jbc.2026.111159)

**Legends**


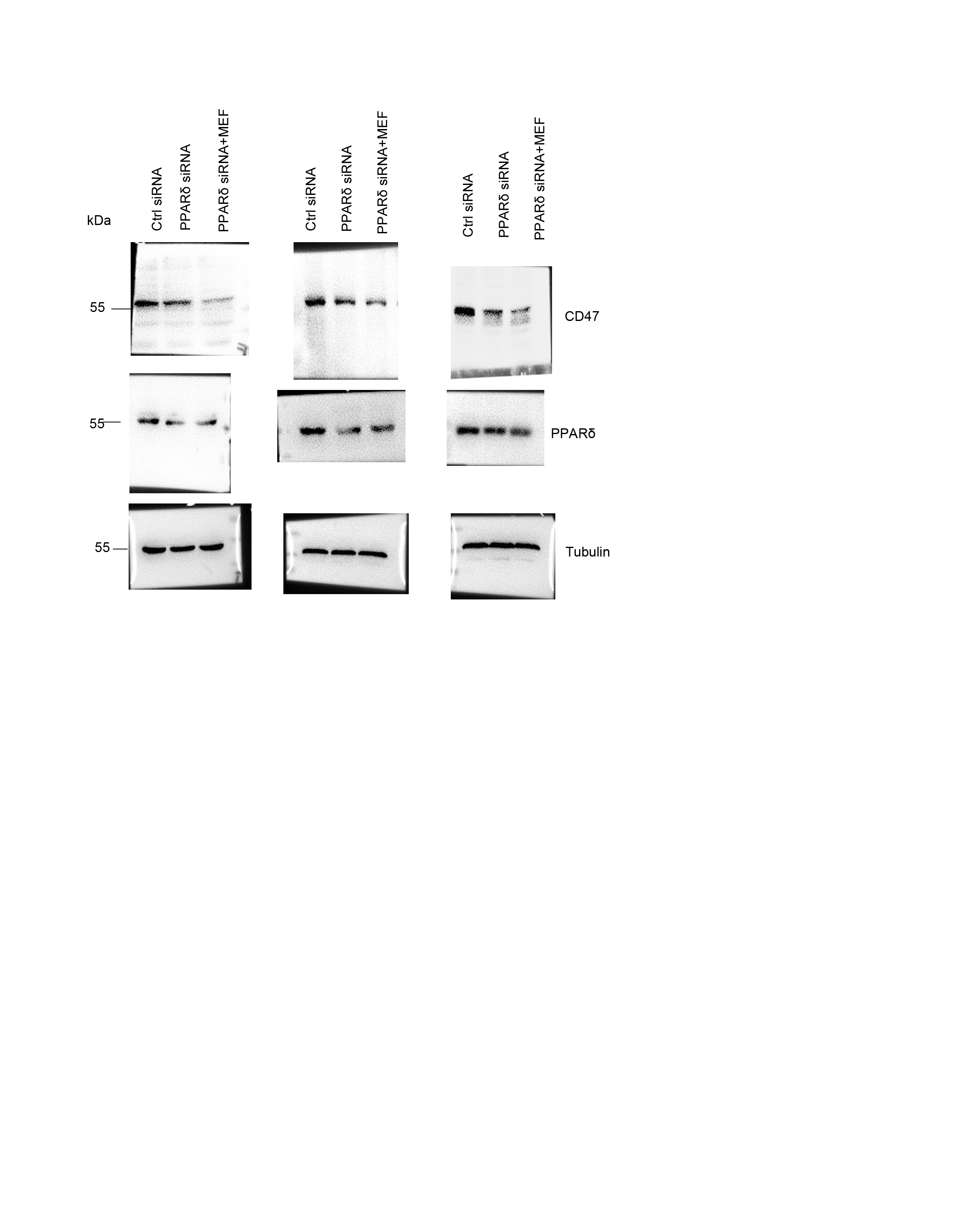


**SFigure. 1.**  **Original scan of Figure 5D.**

Supplement: Supplemental Figure S1 [file mmc1.docx]
